# Transplantation of High Hydrogen-Producing Microbiota Leads to Generation of Large Amounts of Colonic Hydrogen in Recipient Rats Fed High Amylose Maize Starch

**DOI:** 10.3390/nu10020144

**Published:** 2018-01-29

**Authors:** Naomichi Nishimura, Hiroki Tanabe, Erika Komori, Yumi Sasaki, Ryo Inoue, Tatsuro Yamamoto

**Affiliations:** 1 Academic Institute, College of Agriculture, Shizuoka University, 836 Ohya, Suruga-ku, Shizuoka 422-8529, Japan; 2 Department of Nutritional Sciences, Faculty of Health and Welfare Science, Nayoro City University, Kita 8-1, Nishi 4, Nayoro, Hokkaido 096-8641, Japan; htanabe@nayoro.ac.jp (H.T.); e420115@nayoro.ac.jp (E.K.); merci_080@yahoo.co.jp (Y.S.); tyama@nayoro.ac.jp (T.Y.); 3 Department of Agricultural and Life Sciences, Kyoto Prefectural University, 1-5 Hangi-cho, Shimogamo, Sakyo-ku, Kyoto 606-8522, Japan; r-inoue@kpu.ac.jp

**Keywords:** hydrogen, resistant starch, microbiota transplantation, antioxidant effect, rats

## Abstract

The hydrogen molecule (H_2_), which has low redox potential, is produced by colonic fermentation. We examined whether increased H_2_ concentration in the portal vein in rats fed high amylose maize starch (HAS) helped alleviate oxidative stress, and whether the transplantation of rat colonic microbiota with high H_2_ production can shift low H_2_-generating rats (LG) to high H_2_-generating rats (HG). Rats were fed a 20% HAS diet for 10 days and 13 days in experiments 1 and 2, respectively. After 10 days (experiment 1), rats underwent a hepatic ischemia–reperfusion (IR) operation. Rats were then categorized into quintiles of portal H_2_ concentration. Plasma alanine aminotransferase activity and hepatic oxidized glutathione concentration were significantly lower as portal H_2_ concentration increased. In experiment 2, microbiota derived from HG (the transplantation group) or saline (the control group) were orally inoculated into LG on days 3 and 4. On day 13, portal H_2_ concentration in the transplantation group was significantly higher compared with the control group, and positively correlated with genera *Bifidobacterium*, *Allobaculum*, and *Parabacteroides*, and negatively correlated with genera *Bacteroides*, *Ruminococcus*, and *Escherichia*. In conclusion, the transplantation of microbiota derived from HG leads to stable, high H_2_ production in LG, with the resultant high production of H_2_ contributing to the alleviation of oxidative stress.

## 1. Introduction

Many bacteria—estimated at around 10^13^–10^14^—reside in the large intestine, where they have important effects on human health. Most of the bacteria are helpful, but some are harmful. Due to the low redox potential in the large intestine, many of the bacteria are anaerobes: oxygen is not used as the final electron and hydrogen acceptor. Fermentation products such as succinate, lactate, and short chain fatty acids (SCFA) act as the electron and hydrogen acceptors. A byproduct of the fermentation is hydrogen gas, some of which is excreted in the breath and flatus [1]. H_2_ is a stable, non-reactive product, which was classically thought to have no effect in the body. However, Osawa et al. [2] demonstrated that H_2_ gas has a specific antioxidant effect on hydroxyl radicals.

Excess oxidative stress, induced by perturbation of the redox balance, triggers many diseases, such as diabetes, cardiovascular disease, and chronic kidney disease. Therefore, it is important to control redox balance using foods containing antioxidants, such as ascorbic acid, α-tocopherol, and polyphenols. H_2_ could play an important role as an antioxidant, participating in the inhibition of some diseases, such as diabetes [3] and cardiovascular diseases [4]. We demonstrated that colonic H_2_ derived from various non-digestible saccharides, such as high amylose maize starch (HAS) [5], pectin [5], fructooligosaccharides [6], and inulin [6], suppressed hepatic oxidative stress and adipose inflammation in rats. Colonic H_2_, derived from fermentable, non-digestible saccharides, could help supply large amounts of H_2_ for 24 h, and maintain an appropriate redox balance in the liver compared with H_2_ water, because it is not easy to deliver large amounts of H_2_ in the body by the administration of H_2_ water in a continuous manner. However, the amount of H_2_ generated by colonic microbes varies, because colonic microbiota in humans [7] and in rats [5] differ among individuals.

Net H_2_ production is determined by the balance between H_2_-producing and H_2_-utilizing microbes in the large intestine [8]. The microbiota of laboratory animals have been reported to differ among breeders and breeding colonies [9], and fermentation patterns may vary, even with the administration of the same fermentation substrate. The differences would be dependent on maternal microbiota in the breeder and colony. During our preliminary experiments, H_2_ concentration in the portal vein increased only slightly in rats, even after the administration of HAS. These rats were assumed to be low H_2_-generating (LG). The production of different amounts of H_2_ among individual rats is suggested to be dependent on colonic microbiota. The formation of high H_2_-producing microbiota could provide a preventive effect against certain diseases, because microbiota derived from high H_2_-generating rats (HG) could contribute to the alleviation of oxidative stress. Recently, investigators have attempted to change the inherent colonic microbiota in humans [10] and rodents [11] by fecal microbiota transplantation in order to inhibit certain diseases.

In the present study, we examined whether different H_2_ production amounts in rats fed HAS affects the alleviation of hepatic oxidative stress in a model of acute hepatic oxidative stress, and whether the transplantation of rat cecal microbiota with high H_2_ production could shift LG to HG.

## 2. Materials and Methods

### 2.1. High Amylose Maize Starch

High amylose maize starch (Hi-maize 1043; total dietary fiber, 64.5% [12]; resistant starch, 45.7% [12], and amylose, 70%) was kindly supplied by Nippon NSC Ltd. (Tokyo, Japan). We previously confirmed, using ileorectostomized rats, that 53% of HAS was digested in the small intestine, and the remainder reached the large intestine [13].

### 2.2. Animals and Diets

This study was approved by the Nayoro City University Animal Use Committee, and animals were maintained in accordance with the Guidelines for the Care and Use of Laboratory Animals, Nayoro City University (approval number 10-01 and 11-04). Eight-week-old, male Sprague-Dawley rats, weighing 210–230 g, were obtained from Japan SLC (Haruno colony or Ohara colony; Shizuoka, Japan). We used rats from the Haruno colony as HG, and those from the Ohara colony as LG, because in our preliminary experiment, we noticed that many rats from the Haruno colony produced high amounts of colonic H_2_, while many rats from the Ohara colony showed low H_2_ production. Rats were housed in individual cages with screen bottoms made of stainless steel, in a room maintained at 23 ± 2 °C, and humidity ranging from 50% to 70%, under lighting conditions of 12 h of light (0700 to 1900) and 12 h of darkness. Rats were acclimated by feeding a lab chow (CE-2, Japan Clea, Tokyo, Japan) for three days in all of the experiments.

### 2.3. Portal H_2_ Concentration and Alleviation of Oxidative Stress among Individual Rats (Experiment 1)

To clarify that even rats fed the same diet produce different amounts of colonic H_2_, and that the difference could affect the alleviation of oxidative stress, we examined whether higher portal H_2_ concentration alleviated oxidative damage in rats after a hepatic ischemia–reperfusion (IR) operation. After the acclimation period, 66 rats (33 rats from the Haruno colony and 33 from the Ohara colony) were fed a diet supplemented with 200 g of HAS per kg for 10 days. The composition of the HAS diet was as described previously [5]. At the end of the feeding period, all of the rats underwent a hepatic IR operation (ischemia time, 30 min; reperfusion time 45 min) under anesthesia, as described previously [5]. Briefly, under pentobarbital sodium anesthesia (70 mg/kg body weight ip), the hepatic artery and portal vein to the left lateral and median lobe was occluded using a bulldog clamp for 30 min while allowing blood flow through the remaining sections. The clamps were removed after ischemia, and hepatic reperfusion was initiated. Rats were killed 45 min after reperfusion by exsanguination under anesthesia. During surgery, the abdominal incision site was wrapped in plastic wrap to prevent tissues from drying out. Rats were placed over an isothermal pad to maintain their body temperature at 37 °C.

### 2.4. Sampling

At the end of the experimental period, 1 mL of blood from the portal vein was successively collected into sealed heparin vials and microtubes, under anesthesia (pentobarbital 50 mg/kg body weight), for H_2_ analysis and plasma preparation, respectively. After the incubation for 1 min at 37 °C, a 1 mL sample of the gaseous phase was withdrawn using a gas-tight syringe, and H_2_ concentration was determined using a gas chromatography (GC) (lower detection limit, 0.10 ppm; quantification range, 0.30–50 ppm; Biogas analyzer BAS-1000; Mitleben, Osaka, Japan). The remaining blood sample was separated by centrifugation (1200× *g* for 20 min at 4 °C), and plasma samples were stored at −80 °C until alanine aminotransferase (ALT) analysis. After blood withdrawal, the liver was perfused immediately with 20 mL cold saline at 4 °C via the portal vein. Immediately after perfusion, the median lobe (ischemic area) was removed, and a portion was rapidly frozen in liquid N_2_. Samples were stored at −80 °C until the glutathione assays. The cecum was removed and weighed, and the cecal content was collected and stored at −80 °C in air-tight tubes until SCFA analysis and bacterial DNA analysis. The cecal tissue was blotted and weighed after rinsing in an ice-cold saline solution.

### 2.5. Assessment of Oxidative Stress and Damage in the Liver

Hepatic reduced glutathione (GSH) and oxidized glutathione (GSSG) levels were determined using the method of Anderson [14] and Raman et al. [15]. Briefly, 1 volume of liver tissue was homogenized in 9 volumes of 5% 5-sulfosalicylic acid, and centrifuged (10,000× *g* for 5 min at 4 °C). The supernatant was used in the 5,5′-dithiobis(2-nitrobenzoic acid)-glutathione reductase recycling assay to determine total glutathione and GSSG concentrations. GSH concentration was calculated from the difference between total glutathione and GSSG. Plasma ALT activity was measured using a commercial kit, Transaminase CII-test (Wako Pure Chemical Industries, Tokyo, Japan), according to the manufacturer’s instructions. 

### 2.6. SCFA Analysis

After homogenization of the cecal contents, cecal organic acids were measured using an HPLC system LC-10A (Shimadzu, Kyoto, Japan) equipped with a Shim-pack SCR-102H column, 8 mm i.d. 30 cm long (Shimadzu, Kyoto, Japan) and an electroconductivity detector CDD-6A (Shimadzu, Kyoto, Japan) [16]. Briefly, 300 mg cecal content was homogenized in 2 mL of 10 mmol/L NaOH containing 0.5 g/L crotonic acid as an internal standard, and then centrifuged at 10,000× *g* at 4 °C for 15 min. The supernatant was subjected to HPLC analysis after deproteinization with an equal volume of chloroform.

### 2.7. Preparation of Inoculum from the Cecal Contents of High H_2_-Generating Rats (Experiment 2)

We prepared cecal microbiota derived from HG fed the HAS diet. After the acclimation period, 12 rats (eight rats from the Haruno colony, and four from the Ohara colony) were given a diet supplemented with 200 g of HAS per kg for 7 days. At the end of the feeding period, portal H_2_ concentration was immediately measured in rats under anesthesia (pentobarbital 50 mg/kg body weight) using a GC analyzer, Biogas analyzer BAS-1000 (Mitleben, Osaka, Japan). Using the portal H_2_ concentration data, the three rats with the highest portal H_2_ concentration (>7.4 μmol/L) were selected as the HG, while the two rats with the lowest portal H_2_ concentration (<2.0 μmol/L) were selected as the LG. Rats in between were treated as the middle H_2_-generating rats. Rats were killed by exsanguination under anesthesia. The cecum was then immediately removed and weighed, and the cecal content was collected under a CO_2_ gas stream. SCFA concentration and the number of total anaerobes were assayed in each rat’s cecal contents. Cecal contents collected from HG were pooled, and then diluted 1 to 10 with pre-reduced sterile dilution buffer (pH 7.2) containing 4.5 g/L KH_2_PO_4_, 6.0 g/L K_2_HPO_4_, 0.5 g/L l-cysteine hydrochloride monohydrate and 0.5 g/L Tween 80 [17]. The suspension was centrifuged (30× *g*, 2 min, 4 °C) to remove debris, and the upper layer was collected as rat microbiota. Sterile glycerol was added to the upper layer to produce a 10% final glycerol concentration. The total anaerobe count, measured using the culture method described below, was 1.8 × 10^9^ cfu/mL. The inoculum was dispensed into a vial, immediately frozen in liquid nitrogen, and stored at −80 °C until further use. The total anaerobe count in the inoculum was verified before inoculation into rats to check that the bacterial count had not been adversely affected by the storage procedure. The preparation was performed under a CO_2_ gas stream, and pre-reduced solutions, which were deoxygenated with CO_2_, were used. The same method was also used on the cecal contents from LG for comparison. Microbiota compositions were analyzed in the two inoculum, using the method described below.

### 2.8. Effects of Transplantation of HG Cecal Microbiota into LG on Portal H_2_ Concentration (Experiment 2)

We determined whether transplanting rat cecal microbiota derived from HG changed H_2_ production and cecal microbiota in LG. After the acclimation period (day 0), 21 rats (Ohara colony) were divided into two groups based on body weight, and all of the rats were given a diet supplemented with 200 g of HAS per kg for 13 days. All of the rats were administered omeprazole (50 mg/kg/day) through oral gavage for 3 days (days 0, 1 and 2) at the start of the experimental period to inhibit the secretion of gastric acid. After omeprazole administration, the inoculum (1.8 × 10^9^ cfu/rat/day; 1 mL) was administered for 2 days (days 3 and 4) through oral gavage to the transplantation group (*n* = 11), according to the method of Manichanh et al. [18]. Sterile, pre-reduced saline was given to the control group (*n* = 10) using the same inoculum volume. H_2_ excretion (Breath + flatus) was measured using a GC analyzer (Biogas analyzer BAS-1000), as described in our previous study [5,6]. H_2_ excretion was determined on days 0, 3, 6, 10, and 13.

### 2.9. Total Anaerobe Count in the Inoculum and Cecal Contents (Experiments 2)

The cecum was obtained immediately after the collection of portal blood for analysis of H_2_. An aliquot of the cecal contents was immediately collected in pre-weighed sterile tubes under a CO_2_ gas stream, and weighed. Nine volumes of pre-reduced dilution buffer (pH 7.2), containing 6 g/L Na_2_HPO_4_, 4.5 g/L KH_2_PO_4_, 0.5 g/L l-cysteine hydrochloride monohydrate and 0.5 g/L Tween 80 [17], was added to the cecal contents before homogenization. Serial 10-fold dilutions of the homogenates were prepared from each sample. Aliquots (100 μL) of each diluent were applied to pre-reduced glucose blood liver (BL) agar (Nissui Pharmaceutical, Tokyo, Japan) as a non-selective medium for anaerobes. The plates were incubated anaerobically at 37 °C for 48 h before the bacterial colonies were counted.

### 2.10. Bacterial DNA Extraction from the Inoculum and the Cecal Content (Experiments 2)

Bacterial DNA from the two inoculum and the cecal contents was isolated from frozen cecal contents using a DNA extraction kit (ISOFECAL for Beads Beating kit, Nippon Gene, Tokyo, Japan), according to the manufacturer’s protocol, except for sample disruption. Frozen cecal contents (200 mg) were disrupted at 2700 rpm for 90 s using a Multi-Beads shocker (MB601U, Yasui Kikai, Osaka, Japan). Extracted DNA samples were dissolved in DNase-free Tris-EDTA buffer (pH 8.0; 10 mmol/L Tris-HCl, 1 mmol/L EDTA), and stored at −30 °C until 16S rDNA sequencing analysis.

### 2.11. Library Preparation and DNA Sequencing

First, 16S rDNA sequencing using a MiSeqTM system (Illumina, San Diego, CA, USA) was conducted according to a previously described method [19]. PCR amplification of the 16S rRNA V3–V4 region was conducted using primers 341F and 805R. The processing of sequencing data, including quality filtering, chimera check, operational taxonomic unit (OTU) definition, and taxonomy assignment was carried out using the Quantitative Insights Into Microbial Ecology (QIIME) [20], USEARCH (https://www.drive5.com/usearch/) [21], and UCHIME (https://www.drive5.com/usearch/manual/uchime_algo.html) [22], exactly as previously described [19].

### 2.12. Statistical Analysis

A power analysis was performed with G Power version 3.1 to determine an adequate sample size for studying a significant difference between portal H_2_ concentration and plasma ALT activity as the primary outcome. The sample size was calculated using the power procedure for Student’s *t*-test, considering an α probability of 0.05 with a power of 0.80, and an effect size was calculated using the results from our previous study [5]. This power analysis determined that a sample size of 13 to 14 rats (experiment 1) and 10 to 11 rats (experiment 2) per group was required for studies. Values obtained from the experiments were expressed as means, with their standard errors or medians with range. In experiment 1, rats were categorized into quintiles of portal H_2_ concentration at the end of the experiment. The number of rats from the respective colony in quintiles 1 to 5 was analyzed using Pearson’s Chi-square test. The Jonckheere–Terpstra trend test was used to assess the effect of colonic H_2_ production on oxidative stress and damage. The medians of quintiles 2 through to 5 were compared with those of quintile 1 using the Steel test. In other experiments, data were subjected to Bartlett’s test for homogeneity of variances, and data with unequal variances were log transformed. For samples with equal variances, Student’s *t*-test was used for comparisons between individual group means. If sample variances were still unequal after log transformation, we used Welch’s *t*-test. Then, α-diversity metrics (Chao1 index, Shannon index and Observed OTU index) were computed, β-diversity metrics were measured using unweighted and weighted UniFrac, and principal coordinate analysis was performed based on the UniFrac distances. The difference of sample clustering was analyzed by permutational multivariate analysis of variation (PERMANOVA). The relationship between portal H_2_ concentration and the population of bacteria was determined by Spearman’s rank correlation coefficient. The statistical analyses, except for the Jonckheere–Terpstra trend test and PERMANOVA, were performed using SAS JMP software (version 9.0.2; Tokyo, Japan). The Jonckheere–Terpstra trend test and PERMANOVA were performed using R software (version 3.2.3, http://www.r-project.org; R Foundation for Statistical Computing, Vienna, Austria) and QIIME (version 1.9.0, http://qiime.org/), respectively. Significance was defined as *p* < 0.05.

## 3. Results

### 3.1. Experiment 1

We examined the relationship between colonic H_2_ production and hepatic oxidative damage. Rats were categorized into quintiles of portal H_2_ concentration at the end of the experiment (Table 1). In quintiles 4 and 5, which had the highest portal H_2_ concentrations, more than 90% of the rats were from the Haruno colony. Portal H_2_ concentrations in quintiles 4 and 5 were 5.5 and 11.3 times that of quintile 1, respectively. Food intake, HAS intake, and body weight gain did not differ among the quintiles. Plasma ALT (alanine aminotransferase) activity was significantly lower when the portal H_2_ concentration was higher, and it was significantly lower in quintile 3 compared with quintile 1. Hepatic GSSG (reduced glutathione) concentration was significantly lower when the portal H_2_ concentration was high. In addition, the GSH (oxidized glutathione)/GSSG ratio tended to be higher (*p* = 0.0928) when the portal H_2_ concentration was high. Quintiles with high portal H_2_ concentration showed higher cecal acetate and butyrate concentrations, and lower cecal propionate and succinate concentrations. These differences in organic acids were statistically significant between quintiles 4 and 5, and quintile 1.

### 3.2. Experiment 2

The portal H_2_ concentration in rats used to prepare the inoculum for the transplantation experiment was 9.27 μmol/L (Supplementary Materials Table S1). The population of microbiota in the prepared inoculum differed between HG and LG. The phylum-level microbial community structure of the high H_2_ inoculum was 34.0% *Bacteroidetes*, 24.8% *Firmicutes*, 33.0% *Actinobacteria*, 3.7% *Proteobacteria*, and 4.5% *Verrucomicrobia*. The population of the phyla *Bacteroidetes* and *Actinobacteria* in the inoculum derived from HG were 0.5 and 1.2 times that of the population from LG, respectively (Supplementary Materials Table S2).

We examined whether the transplantation of colonic microbiota derived from HG could shift LG to HG (Table 2). No significant difference was observed in food intake and body weight gain. Until day 6 (2 days after final inoculation), low H_2_ excretion (breath + flatus) was maintained in both groups. On and after day 10, the H_2_ excretion in the transplantation group was more than twice the value of the control group, although no significant difference was observed. Portal H_2_ concentration was significantly higher in the transplantation group compared with the control group, and was comparable to that in donor rats (Supplementary Materials Table S2). The cecal weight of content and tissue, and concentrations of propionate and butyrate did not differ between the groups. Cecal acetate concentration was significantly higher in the transplantation group compared with the control group. Cecal succinate concentration tended to be lower in the transplantation group compared with the control group (*p* = 0.0701).

**Figure 1:**
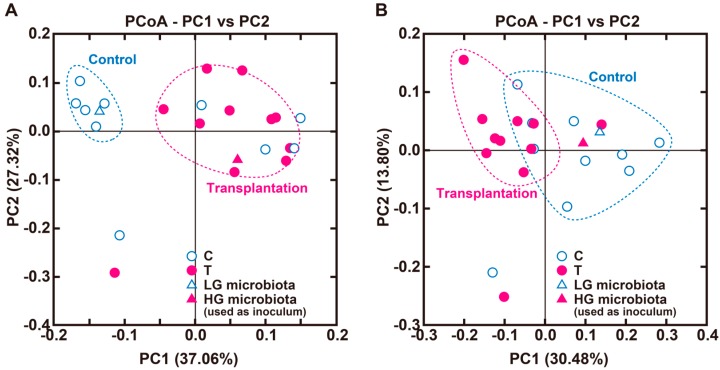
Principle coordinate analysis plot based on weighted (**A**) and unweighted (**B**) UniFrac distances of microbial communities in the cecum of control and transplantation rats. Sample clustering was significant between both groups (*p* = 0.086 and *p* = 0.006, respectively, PERMANOVA). control group, *n* = 10; transplantation group, *n* = 11. C, control group; HG, high H_2_ generators; LG, low H_2_ generators; PCoA, principal coordinate analysis; PERMANOVA, permutational multivariate analysis of variation; T, transplantation group.

Cecal anaerobe count did not differ between the groups. For microbiota composition analysis, a total of 312,451 sequence reads were obtained after data processing (average 14,202 reads/sample; standard deviation 10,048). For α-diversity, the Shannon index was significantly lower in the transplantation group than in the control group (control group, 2.47 ± 0.13; transplantation group, 1.97 ± 0.06; *p* = 0.0051), while the Chao1 index and observed OTU index did not differ between the groups (Chao1 index, 72.6 ± 3.1 and 69.5 ± 3.4; *p* = 0.5236; observed OTU index, 63.0 ± 1.9 and 60.4 ± 3.3; *p* = 0.4947 in the control and transplantation groups, respectively). Although microbiota communities tented to be different between both groups (*p* = 0.086; Figure 1) by β-diversity analyses using weighted UniFrac, a specific cluster was formed in the transplantation group that was similar to HG microbiota. β-diversity analyses using unweighted UniFrac revealed that microbiota communities were significantly distant from each other (*p* = 0.006; Figure 1). The cecal population of the *Bacteroidales* family S24-7 and the genera *Oscillospira* and *Escherichia* were significantly greater in the transplantation group compared with the control group, while the genus *Bacteroides* was significantly lower (Table 3). There was no significant difference in the population of the *Desulfovibrionaceae* family, which utilizes H_2_ to produce hydrogen sulfide, between both groups (data not shown), and the populations were less than 0.01% in both groups. The genus *Desulfovibrio*, which belongs to the *Desulfovibrionaceae* family, was not detected. Portal H_2_ concentration positively correlated with genera *Bifidobacterium*, *Parabacteroides*, and *Allobaculum*, and negatively correlated with genera *Bacteroides*, *Ruminococcus*, and *Escherichia*.

## 4. Discussion

We previously found that colonic H_2_ suppressed hepatic oxidative stress and damage in IR rats that were fed either the HAS or pectin diets compared with those fed a control diet [5]. In the present study, liver damage due to a hepatic IR operation was improved in HAS-fed rats with a high portal H_2_ concentration (Table 1). These results suggest that the alleviating effect of colonic H_2_ on hepatic oxidative stress and damage is dependent on portal H_2_ concentration. In our previous study, we screened and used only HG: the portal H_2_ concentrations were 4.5–11 μmol/L, with the high range of portal H_2_ concentration leading to suppressed oxidative stress and damage [5]. The range in the previous study is comparable with the range in quintiles 3 and 4 in the present study (Table 1). Although the difference in the hepatic GSH/GSSG ratio did not reach statistical significance (*p* = 0.0928), hepatic oxidative stress (liver GSSG concentration) and damage (plasma ALT activity) after the IR operation were significantly improved when the portal H_2_ concentration was high: greater than 4.3 μmol/L, which corresponded to quintiles 3–5. Hepatic oxidative damage was significantly improved in quintile 3 compared with quintile 1. Colonization by high H_2_ producing microbiota, as well as the administration of high fermentable, non-digestible saccharides, would be required for a stable supply of large amounts of H_2_ in the body, because fermentation substrate and colonic bacteria cause colonic fermentation.

Net H_2_ production is determined by a balance between H_2_-producing and H_2_-utilizing microbes in the large intestine [8]. Colonic H_2_ is mainly produced by members of the phylum Firmicutes and the phylum Bacteroidetes, and utilized predominantly by reductive acetogens, sulfate-reducing bacteria, and methanogens to produce acetate, hydrogen sulfide, and methane, respectively [8,23]. Since the microbiota of laboratory animals also differ among the breeders and breeding colonies depending on maternal microbiota [9], fermentation patterns would vary, even with the administration of the same fermentation substrate. In the present study, we observed large differences in portal H_2_ concentration, which is a marker of colonic H_2_ production, among the colonies of the same breed or individuals in the same colony. Therefore, the different H_2_ productions in the present study reflect the composition of colonic microbiota. Manichanh et al. showed that the transplantation of cecal microbiota to rats without antibiotic treatment produced a similar composition of microbiota between the transplanted rats and the donor rats [18]. Using the same method, we determined that the transplantation of microbiota derived from HG into LG caused high H_2_ production in the transplanted rats (Table 2 and Figure 1). Previous investigators reported that bacteria used as probiotics did not colonize the large intestine over the long term unless the bacteria were continuously administered [24]. Although the shift by transplantation may be temporary, this result shows that the presence of high H_2_-producing microbiota in the large intestine causes an increase in H_2_ production. In the present study, we used rat cecal microbiota as the inoculum. Li et al. demonstrated successful fecal microbiota transplantation in humans [25]. The results suggest that the microbiota colonization, using the same species of animal, might be relatively easy; however, colonization using a single species of bacteria may not be successful.

Inoculum was prepared from rats showing high H_2_ production in their large intestine. In comparison with the microbiota from LG, the high H_2_-producing inoculum showed a higher population of the phylum *Actinobacteria* (predominantly bifidobacteria) and lower population of the genus *Bacteroides*. The differences were maintained in the cecal content of rats transplanted with the inoculum (Supplementary Materials Figure S2); populations of the phyla Actinobacteria and Bacteroidetes in the transplantation group were 153% and 69% higher than in the control group, respectively. HAS containing resistant starch selectively promotes *Bifidobacterium* spp. according to its availability [26,27]. HAS is strongly utilized by a limited type of bacteria, such as *Bifidobacterium* spp. [27,28,29], but not *Bacteroides* spp. [28]. The change in these bacteria, due to transplantation, is suggested to be reasonable to promote the utilization of HAS. Bifidobacteria cannot produce H_2_ gas [23,30], because they do not have hydrogenase, although they can degrade resistant starch to produce acetate and lactate. Lactate is utilized by other bacteria to produce acetate and butyrate [31]. Therefore, even though bifidobacteria cannot produce H_2_ gas, a 50% increase in the population of the genus *Bifidobacterium* in the transplantation group could promote the colonic fermentation of HAS and stimulate high H_2_ production through metabolite cross-feeding by any type of H_2_-producing bacteria [8,32,33]. It would be difficult to identify the H_2_-producing bacteria involved in high portal H_2_ concentration due to transplantation, because 66% of the total bacteria in human feces are estimated to produce H_2_ [23]. Although not all of the bacteria in the inoculum reached the large intestine of the recipient rats alive and colonized, we suggest that bacteria contributing to the increased H_2_ production came from the inoculum, and were successful in colonizing the large intestine. In the present study, the higher H_2_ quintiles and the transplantation group showed higher colonic acetate concentration. These results could reflect the finding that H_2_-producing bacteria convert pyruvate to acetate, carbon dioxide, and H_2_ [34]. However, H_2_ is also produced by other reactions. Moreover, H_2_ and acetate are utilized by some bacteria to produce other metabolites, such as methane and butyrate [34]. Therefore, further investigation need to be performed to determine relationships between colonic production of H_2_ and organic acids.

In the present study, sulfate-reducing bacteria, such as the Desulfovibrionaceae family, which consumed H_2_ to produce hydrogen sulfate, were detected at very low levels. A negative correlation between portal H_2_ concentration and the genus *Ruminococcus*, part of which includes reductive acetogens, were observed after the transplantation of inoculum. These results suggest that some H_2_-utilizing bacteria may be related to the supply amount of H_2_ in the body. However, methanogens, which is H_2_-utilizing archaea, could not be detected, because PCR amplicon of the 16S rRNA V3–V4 region, which was conducted using primers 341F and 805R, was sequenced. Low H_2_ partial pressure is essential to maintain anaerobic fermentation, because high H_2_ partial pressure inhibits reduced nicotinamide adenine dinucleotide dehydrogenase and ATP production [35,36]. Therefore, excess accumulation of H_2_ might be inadequate in regards to the continuing fermentation and proliferation of bacteria. The extent of colonic H_2_ production that can alleviate oxidative stress without inhibiting fermentation remains unclear. Moreover, changes in H_2_ concentration may also result in changes in microbiota, because high H_2_ partial pressure inhibits proliferation of some anaerobes. Further investigation is required to overall verify the physiological significance of high H_2_ production and mechanisms by which transplantation of high H_2_ inoculum could lead to a high amount of net H_2_ production in rats that have been fed HAS. In the present study, transplanted microbiota colonized for 10 days after the final inoculation, and changes in the abundance of the phyla and genera due to transplantation, caused high H_2_ production. However, it remains unclear whether transplanted microbiota would continue to colonize the large intestine in recipients over longer periods, and continue to produce large amounts of H_2_. Further study is required to study the long-term effect of transplantation on portal H_2_ concentration.

In conclusion, portal H_2_ concentration differs widely among individual rats, even when the same non-digestible saccharides were fed. H_2_ production was determined to be dependent on the composition of cecal microbiota, which varies between individual rats. Furthermore, we found that LG converted to HG after the transplantation of microbiota derived from HG. High portal H_2_ concentration correlated with greater suppression of oxidative stress. The formation of an appropriate composition of colonic microbiota, and the supply of suitable fermentation substrate to the large intestine, would lead to high colonic H_2_ production, which may contribute to the alleviation of acute oxidative stress induced by hepatic IR operation.

## Figures and Tables

**Table 1:** Liver redox status, plasma alanine aminotransferase (ALT) activity, and cecal organic acid concentration, according to the quintiles of rat portal H_2_ concentration.

	Q1 (Low)	Q2	Q3	Q4	Q5 (High)	χ^2^ (*p*)
Haruno colony	4	1	3	12	13	<0.0001
Ohara colony	9	12	10	1	1	<0.0001
						P_trend_
Portal H_2_, μmol/L	1.54 (0.771–1.89)	3.39 (2.75–3.92)	6.08 ** (5.02–6.56)	8.45 *** (8.02–9.06)	17.4 *** (13.2–19.9)	<0.0001
Food intake, g/10 day	198 (190–204)	192 (182–208)	199 (188–206)	202 (187–209)	196 (190–200)	0.4594
HAS intake, g/10 day	39.6 (38.1–40.9)	38.3 (36.4–41.7)	39.7 (37.7–41.3)	40.3 (37.5–41.9)	39.2 (37.9–40.0)	0.4752
Body weight gain, g/10 day	70 (61–85)	78 (68–84)	72 (64–77)	72 (66–78)	71 (60–82)	0.2687
Liver, μmol/g tissue						
GSSG	0.137 (0.111–0.157)	0.171 * (0.139–0.185)	0.157 (0.143–0.174)	0.145 (0.123–0.159)	0.122 (0.106–0.135)	0.0314
GSH	7.20 (6.77–7.88)	7.13 (6.92–7.93)	7.22 (6.91–8.40)	6.83 (6.31–7.30)	7.01 (6.74–7.25)	0.1087
GSH/GSSG	55.4 (46.4–69.5)	45.9 (40.1–48.6)	47.9 (40.8–55.2)	48.1 (42.2–58.2)	59.6 (50.8–67.2)	0.0928
Plasma ALT, μkat/L	7.96 (3.84–8.90	6.33 (0.717–9.43)	3.54 * (0.526–4.92)	2.03 (0.582–7.88)	3.99 (0.750–9.88)	0.0422
Cecal organic acid, μmol/g						
Acetate	42.1 (37.4–59.3)	41.6 (35.8–43.4)	47.2 (33.3–60.6)	71.4 * (60.0–78.5)	87.7 *** (66.0–97.0)	<0.0001
Propionate	8.77 (8.01–10.3)	11.4 (7.90–15.3)	8.75 (5.60–10.0)	4.13 ** (3.32–6.23)	4.83 ** (3.59–5.55)	<0.0001
Butyrate	4.79 (2.97–6.84)	4.25 (3.56–5.88)	6.60 (3.85–9.64)	9.34 * (7.15–12.1)	12.3 *** (10.3–15.7)	<0.0001
Succinate	60.8 (38.9–69.3)	45.3 (41.6–48.5)	45.4 (27.5–51.2)	25.8 ** (18.8–35.0)	30.7 ** (24.9–37.0)	<0.0001

Data are expressed as the medians (25%–75%), *n* = 13 or 14. All of the rats were fed high amylose maize starch for 10 days, and then underwent ischemia–reperfusion (IR) treatment (30 min of ischemia + 45 min of reperfusion) at the end of the experiment. Rats were categorized into quintiles of portal H_2_ concentration at the end of the experiment. Data were analyzed using the Jonckheere–Terpstra trend test (all of the parameters except for the number of rats) and Steel test (all of the parameters except for the number of rats), or the Chi-square test (the number of rats). *, **, *** Median values were significantly different from those of the Q1 (*p* < 0.05, *p* < 0.01, *p* < 0.001) quintile; HAS, high amylose maize starch; GSH, reduced glutathione; GSSG, oxidized glutathione.

**Table 2:** Changes in body weight, food intake, cecal H_2_, organic acid production, and cecal counts of total anaerobes in HAS-fed rats transplanted with high H_2_-producing microbiota.

	Control	Transplantation	*p* Value
Initial body weight (g on day 0)	238 ± 2	237 ± 2	0.9753
Body weight gain (g/13 day)	87 ± 4	78 ± 4	0.1348
Food intake (g/13 day)	287 ± 7	261 ± 8 *	0.0493
Net H_2_ excretion (μmol/5 min)			
Day 0	0.103 (0.064–0.338)	0.146 (0.101–0.176)	0.8931
Day 3	0.579 (0.447–1.13)	0.405 (0.196–0.830)	0.1690
Day 6	0.334 (0.212–0.679)	0.304 (0.151–0.622)	0.8786
Day 10	0.554 (0.199–0.878)	1.13 (0.252–2.92)	0.1734
Day 13	0.721 (0.347–3.55)	2.34 (1.65–2.96)	0.3910
AUC_day 6–13_ (mmol)	0.966 (0.665–2.64)	3.04 (1.41–6.06)	0.1627
Portal H_2_ (μmol/L)	3.07 ± 1.00	9.95 ± 1.78 **	0.0041
Cecum (g)			
Contents	9.31 ± 0.58	9.59 ± 0.61	0.7443
Tissue	1.87 ± 0.11	1.81 ± 0.10	0.7253
Cecal organic acids (μmol/g)			
Acetate	43.5 ± 3.3	57.8 ± 2.7 **	0.0038
Propionate	9.80 ± 0.98	8.17 ± 1.07	0.2777
n-Butyrate	5.65 ± 1.05	7.06 ± 0.80	0.2999
Succinate	61.4 ± 4.2	48.5 ± 5.2	0.0701
Cecal bacteria			
Total anaerobes (log_10_cfu/g)	12.7 ± 0.5	12.6 ± 0.3	0.7592

Data are expressed as the means ± standard error (SE) or medians (25%–75%), control group, *n* = 10 and transplantation groups, *n* = 11. Data were analyed using the Student’s *t*-test (data expect for net H_2_ excretion and area under the curve (AUC)) or Welch’s test (data for net H_2_ excretion and AUC). Transplantation of the inoculum was performed on days 3 and 4. Net H_2_ excretion in breath and flatus was measured. AUC was calculated from changes in net H_2_ excretion from day 6 to day 13. *, ** Mean values were significantly different from those of the control group (*p* < 0.05 and *p* < 0.01, respectively).

**Table 3:** Changes in the population of cecal microbiota in HAS-fed rats transplanted with high H_2_-producing microbiota.

					Correlation ^‡^ (vs. Portal H_2_)	
Order	Family	Genus	C (%)	T (%)	r	*p*
*Actinobacteria*			12.0 ± 5.5	18.3 ± 4.4	0.789	4.82 × 10^−^^5^
*Bifidobacteriales*	*Bifidobacteriaceae*	*Bifidobacterium*	12.0 ± 5.4	18.0 ± 4.3	0.791	4.49 × 10^−5^
*Bacteroidetes*			58.0 ± 7.3	39.8 ± 4.1 *	−0.408	0.0757
*Bacteroidales*	*Bacteroidaceae*	*Bacteroides*	40.2 ± 9.0	8.0 ± 3.1 **	−0.507	0.0240
*Bacteroidales*	s24-7		15.7 ± 2.7	30.7 ± 4.7 *	−0.084	0.724
*Bacteroidales*	*Porphyromonadaceae*	*Parabacteroides*	1.7 ± 0.5	1.0 ± 0.4	0.316	0.0190
*Firmicutes*			23.8 ± 3.2	34.3 ± 4.6 ^†^	−0.0992	0.677
*Lactobacillales*	*Lactobacillaceae*	*Lactobacillus*	9.3 ± 3.1	9.1 ± 1.9	0.368	0.111
*Clostridiales*	*Lachnospiraceae*	*Blautia*	1.1 ± 0.2	3.1 ± 0.9	−0.107	0.653
*Clostridiales*	*Lachnospiraceae*	*Clostridium*	1.2 ± 0.3	2.1 ± 0.5	0.383	0.0957
*Clostridiales*	*Lachnospiraceae*	Other	5.8 ± 0.9	1.9 ± 0.8	−0.397	0.0841
*Clostridiales*	*Ruminococcaceae*	*Ruminococcus*	1.1 ± 0.5	6.8 ± 4.9	−0.567	0.0103
*Clostridiales*	*Ruminococcaceae*	*Oscillospira*	1.1 ± 0.1	2.1 ± 0.3 *	−0.0391	0.871
*Clostridiales*	*Ruminococcaceae*	Other	1.2 ± 0.2	0.6 ± 0.1	−0.112	0.678
*Erysipelotrichales*	*Erysipelotrichaceae*	*Allobaculum*	1.2 ± 0.4	6.1 ± 2.5	0.666	1.77 × 10^−3^
*Erysipelotrichales*	*Erysipelotrichaceae*	*Eubacterium*	0.1 ± 0.0	0.1 ± 0.0	−0.405	0.0780
*Proteobacteria*			4.1 ± 1.2	4.4 ± 0.7	−0.370	0.109
*Enterobacteriale*	*Enterobacteriaceae*	*Escherichia*	0.3 ± 0.2	3.1 ± 0.7 **	−0.453	0.0466
*Enterobacteriales*	*Enterobacteriaceae*	Other	3.0 ± 1.3	0.0 ± 0.0	−	−
*Burkholderiales*	*Alcaligenaceae*	*Sutterella*	0.8 ± 0.3	1.3 ± 0.2	0.341	0.141
*Verrucomicrobia*			1.9 ± 1.9	3.1 ± 2.7	−0.383	0.0960
*Verrucomicrobiales*	*Verrucomicrobiaceae*	*Akkermansia*	1.9 ± 1.9	3.1 ± 2.7	−0.383	0.0960

Data are expressed as the means ± SE, *n* = 10 and transplantation groups, *n* = 11. Data that were less than 0.1% in both the control and transplantation groups were omitted from the table. Data were analyzed using the Student’s *t*-test or Welch’s test. Transplantation of the inoculum was performed on days 3 and 4. C, control group; T, transplantation group. *, ** Mean values were significantly different from those of the control group (*p* < 0.05, *p* < 0.01). ^†^ Mean values tended to be higher than those of the control group (*p* = 0.0777). ^‡^ Correlations were determined by Spearman’s rank correlation coefficient.

## Supplementary Materials

The following are available online at http://www.mdpi.com/2072-6643/10/2/144/s1, Figure S1: Phyla level distribution of rat cecal contents in rats transplanted with high H_2_-producing microbiota, Figure S2: Genus level distribution in rat cecal contents after transplantation with high H_2_-producing microbiota, Table S1: H_2_ production and cecal organic acid concentration in rats with different colonic H_2_ producing ability, Table S2: Population of cecal microbiota in the inoculum.
