# Supplementary material for: Transplantation of High Hydrogen-Producing Microbiota Leads to Generation of Large Amounts of Colonic Hydrogen in Recipient Rats Fed High Amylose Maize Starch
Source: Nutrients. 2018 Jan 29;10(2):144. doi: 10.3390/nu10020144 (PMC5852720; doi:10.3390/nu10020144)
Supplement: Supplementary file 1 [file nutrients-10-00144-s001.docx]

**Supplementary Materials**

**Table S1.** H_2_ production and cecal organic acid concentration in rats with different colonic H_2_ producing ability ^1^.

|  | High | Middle | Low |
| --- | --- | --- | --- |
| Haruno colony | 3 | 5 | 0 |
| Ohara colony | 0 | 2 | 2 |
| Initial body weight (g) | 286 (282−286) | 275 (268−283) | 256 (254−258) |
| Body weight gain (g/7d) | 49 (41−54) | 41 (31−48) | 44 (44−44) |
| Net H_2_ excretion (μmol/5 min) | 7.58 (3.75−10.24) | 2.22 (1.36−4.82) | 0.312 (0.223−0.401) |
| Portal H_2_ (μmol/L) | 9.27 (7.40−11.4) | 5.47 (3.77−7.05) | 1.41 (1.24−1.58) |
| **Cecum** (g) |  |  |  |
| Contents ^2^ | 9.08 (7.95−9.16) | 8.70 (4.64−10.5) | 9.42 (7.58−11.3) |
| Tissue | 1.77 (1.66−1.79) | 1.51 (1.36−1.67) | 1.49 (1.37−1.60) |
| **Organic acids** (μmol/g) |  |  |  |
| Acetate | 73.3 (62.2−75.3) | 60.6 (34.5−83.3) | 27.8 (27.4−28.1) |
| Propionate | 5.80 (3.45−7.56) | 5.90 (2.83−12.3) | 10.3 (7.11−13.4) |
| n-Butyrate | 5.07 (4.42−6.76) | 5.83 (4.43−8.43) | 3.69 (2.42−4.96) |
| Succinate | 43.5 (21.6−46.5) | 38.0 (15.4−72.5) | 59.3 (47.1−71.5) |
| **Cecal bacteria** (log_10_cfu/g) |  |  |  |
| Total anaerobes | 13.7 (12.4−14.3) | 12.0 (11.0−14.3) | 13.0 (12.1−13.8) |

^1^ Data are expressed as the medians (minimum−maximum). ^2^ Cecal contents in the top 3 H_2_-generating rats were pooled and used in the preparation of inoculum. High, the highest H_2_-generating rats; Low, the lowest H_2_-generating rats; Middle, the remaining rats, between the High and Low group in H_2_ generation.

**Table S2.** Population of cecal microbiota in the inoculum.

| Order | Family | Genus | High ^1^ | Low |
| --- | --- | --- | --- | --- |
|  |  |  | % | |
| ***Actinobacteria*** |  |  | 33.0 | 2.7 |
| *Bifidobacteriales* | *Bifidobacteriaceae* | *Bifidobacterium* | 33.0 | 2.7 |
| ***Bacteroidetes*** |  |  | 34.0 | 70.8 |
| *Bacteroidales* | *Bacteroidaceae* | *Bacteroides* | 8.7 | 49.9 |
| *Bacteroidales* | s24-7 |  | 24.1 | 18.5 |
| *Bacteroidales* | *Porphyromonadaceae* | *Parabacteroides* | 0.7 | 1.5 |
| ***Firmicutes*** |  |  | 24.8 | 21.8 |
| *Lactobacillales* | *Lactobacillaceae* | *Lactobacillus* | 3.1 | 4.0 |
| *Clostridiales* | *Lachnospiraceae* | *Blautia* | 4.1 | 7.1 |
| *Clostridiales* | *Lachnospiraceae* | *Clostridium* | 0.3 | 0.6 |
| *Clostridiales* | *Lachnospiraceae* | Other | 2.3 | 2.1 |
| *Clostridiales* | *Ruminococcaceae* | *Ruminococcus* | 2.5 | 0.3 |
| *Clostridiales* | *Ruminococcaceae* | *Oscillospira* | 5.9 | 2.0 |
| *Clostridiales* | *Ruminococcaceae* | Other | 5.2 | 2.2 |
| *Erysipelotrichales* | *Erysipelotrichaceae* | *Allobaculum* | 0.5 | 0.7 |
| *Erysipelotrichales* | *Erysipelotrichaceae* | *Eubacterium* | 0.0 | 0.1 |
| ***Proteobacteria*** |  |  | 3.7 | 3.9 |
| *Enterobacteriale* | *Enterobacteriaceae* | *Escherichia* | 0.9 | 1.0 |
| *Enterobacteriales* | *Enterobacteriaceae* | Other | 0.0 | 0.0 |
| *Burkholderiales* | *Alcaligenaceae* | *Sutterella* | 2.6 | 2.2 |
| ***Verrucomicrobia*** |  |  | 4.5 | 0.1 |
| *Verrucomicrobiales* | *Verrucomicrobiaceae* | *Akkermansia* | 4.5 | 0.1 |

^1^ Rat colonic microbiota derived from the top 3 H_2_-generating rats were used as the inoculum. High, rat colonic microbiota derived from the highest H_2_-generating rats; Low, rat colonic microbiota derived from the lowest H_2_-generating rats.


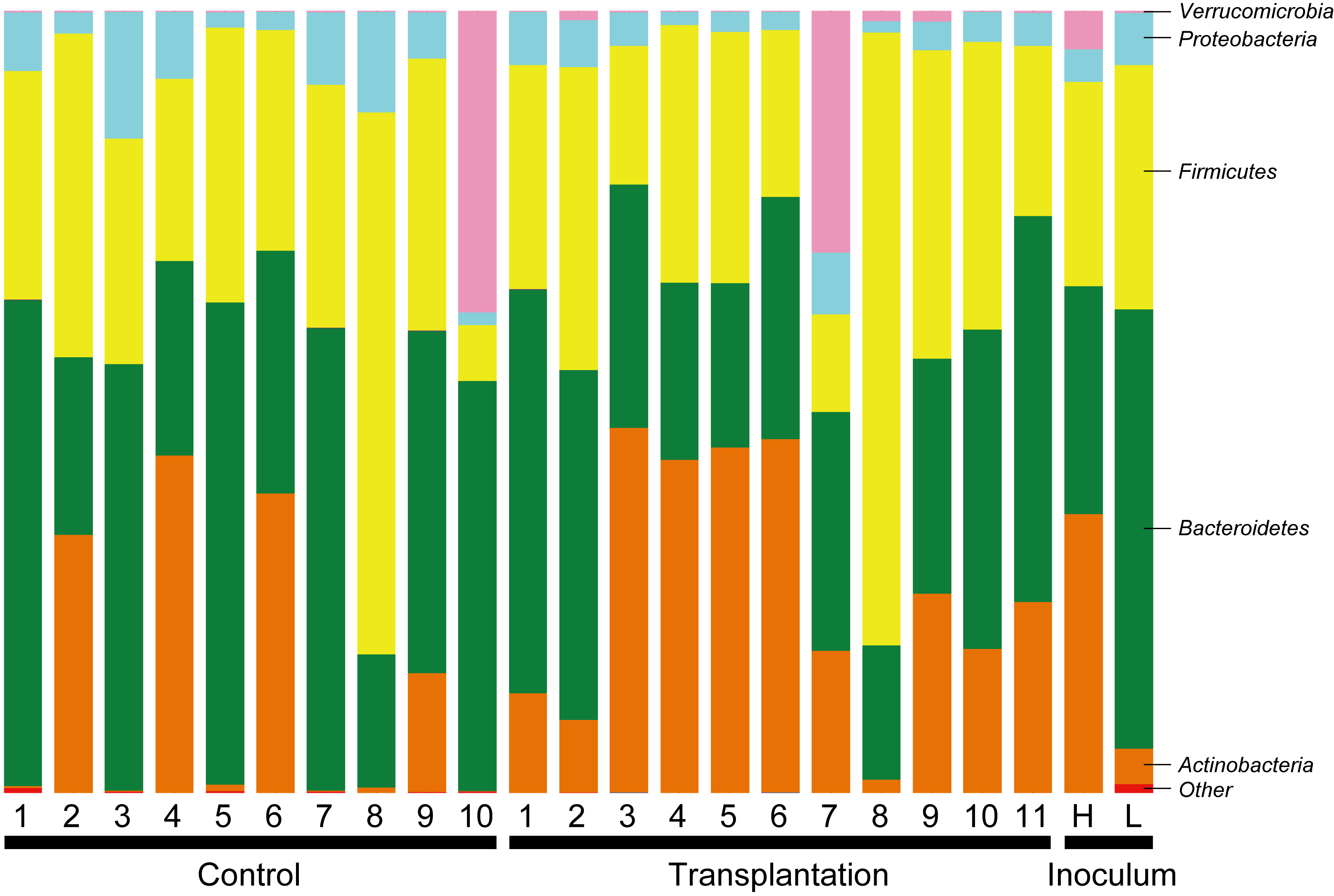

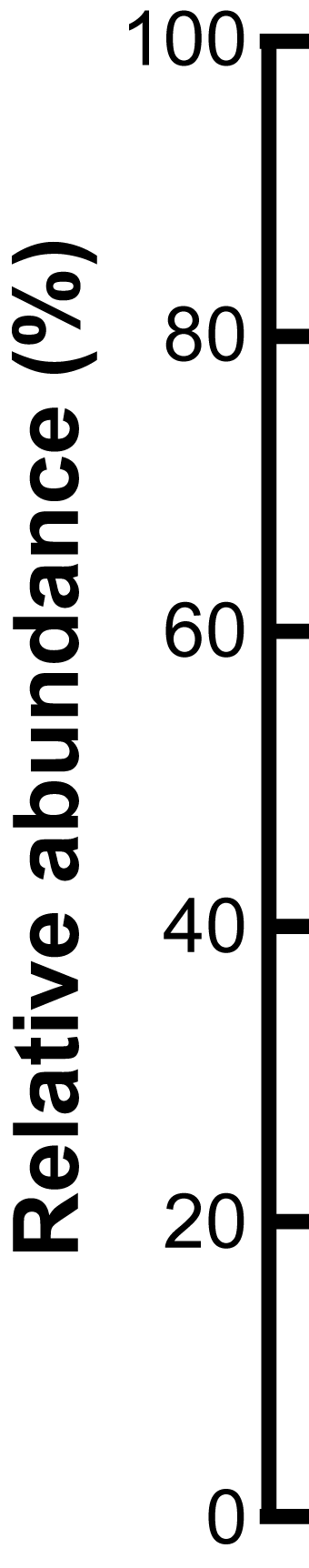


**Figure S1.** Phyla level distribution of rat cecal contents in rats transplanted with high H_2_-producing microbiota. H, inoculum derived from high H_2_-generating rats; L, inoculum derived from low H_2_-generating rats. The number below the bar graph represents individual rats in the control and transplantation groups.


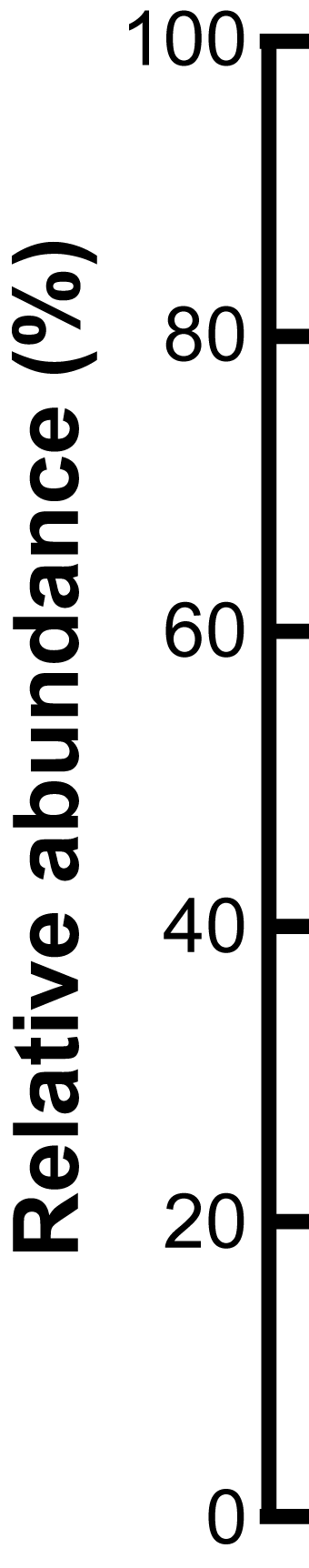

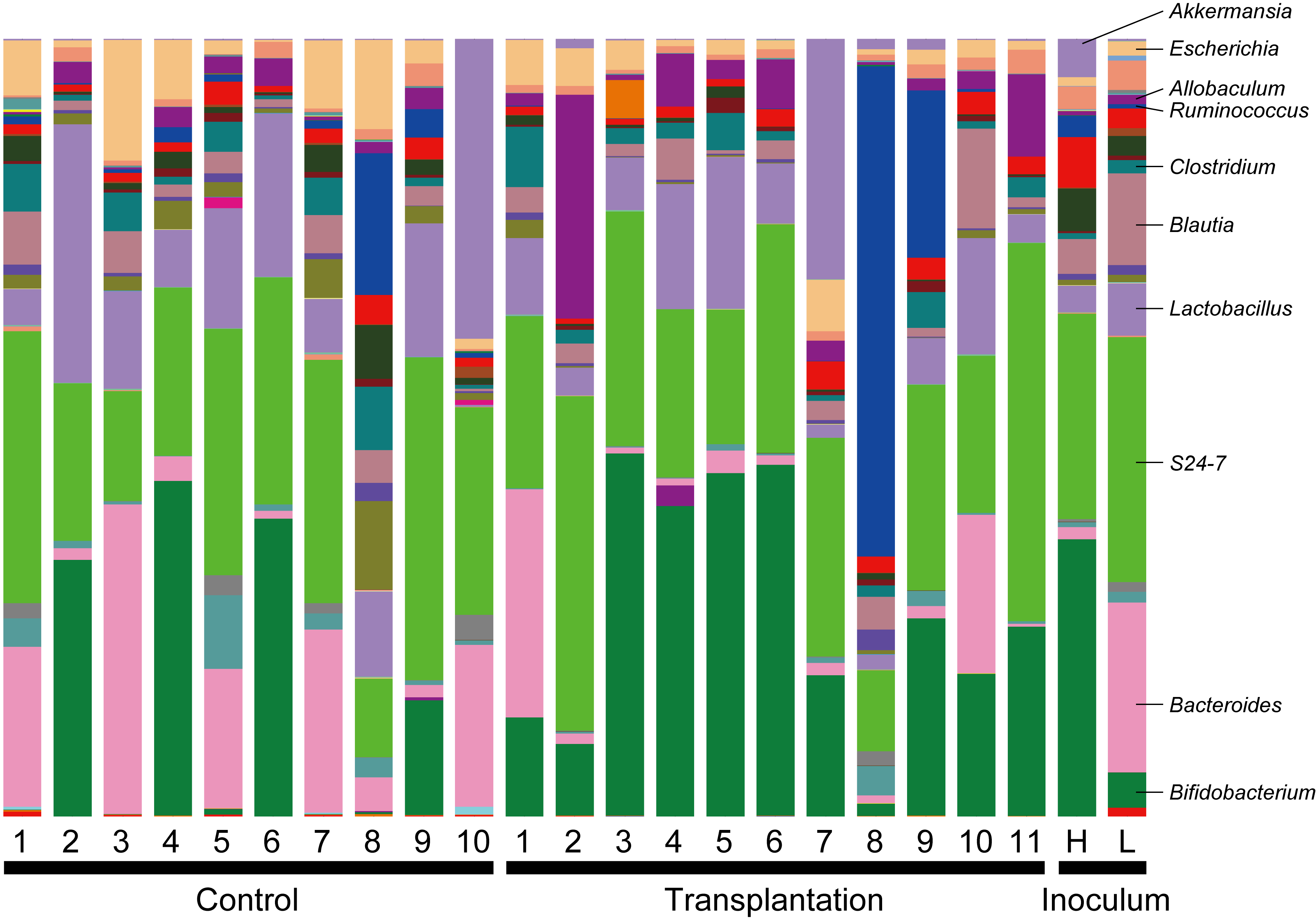


**Figure S2.** Genus level distribution in rat cecal contents after transplantation with high H_2_-producing microbiota. H, inoculum derived from high H_2_-generating rats; L, inoculum derived from low H_2_-generating rats. The number below the bar graph represents individual rats in the control and transplantation groups.
